# Current Views of Toll-Like Receptor Signaling Pathways

**DOI:** 10.1155/2010/240365

**Published:** 2010-12-14

**Authors:** Masahiro Yamamoto, Kiyoshi Takeda

**Affiliations:** Department of Microbiology and Immunology, Graduate School of Medicine and WPI Immunology Frontier Research Center, Osaka University, 2-2 Yamada-Oka, Suita, Osaka 565-0871, Japan

## Abstract

On microbial invasion, the host immediately evokes innate immune responses. Recent studies have demonstrated that Toll-like receptors (TLRs) play crucial roles in innate responses that lead not only to the clearance of pathogens but also to the efficient establishment of acquired immunity by directly detecting molecules from microbes. In terms of intracellular TLR-mediated signaling pathways, cytoplasmic adaptor molecules containing Toll/IL-1R (TIR) domains play important roles in inflammatory immune responses through the production of proinflammatory cytokines, nitric oxide, and type I interferon, and upregulation of costimulatory molecules. In this paper, we will describe our current understanding of the relationship between TLRs and their ligands derived from pathogens such as viruses, bacteria, fungi, and parasites. Moreover, we will review the historical and current literature to describe the mechanisms behind TLR-mediated activation of innate immune responses.

## 1. Introduction

Innate and adaptive immunities play important roles in the elimination of various pathogens such as viruses, bacteria, and parasites in mammals [1–3]. The adaptive immune system exerts highly specific responses to microbes by producing antibodies from B cells or through the generation of killer or helper T lymphocytes, resulting in life-long immunological memory. This process may take weeks, or even months, to establish sufficient levels of immunity. On the other hand, the innate immune system promptly responds to the invasion of microbes and acts as the first line of defense, whereby innate immune cells such as macrophages or dendritic cells (DCs) play a central role in the production of proinflammatory cytokines or nitric oxide. 

Almost 20 years ago, Janeway proposed that pattern recognition receptors (PRRs), which recognize pathogen-associated molecular patterns (PAMPs) specific to each pathogen, are expressed on innate immune cells and discriminate self or nonself structures [4]. However, the existence of PRRs had not been elucidated until 1996, when Hoffman's group identified “Toll” in *Drosophila*, a mutant defective in antifungal defense [5]. Subsequently, a mammalian homolog of Toll was discovered using a bioinformatics approach, and its overexpression in mammalian cells was shown to induce proinflammatory cytokines and induce expression of costimulatory molecules to stimulate acquired immunity [6]. This study opened new avenues for the identification of additional members of this family of proteins, now known as Toll-like receptors (TLRs), in humans and mice [1–3]. To date, more than 10 members of the TLR family have been reported in mammals and function as PRRs, recognizing a variety of PAMPs, such as lipopolysaccharide, lipoprotein, nucleic acids, amongst others [1–3]. 

Signaling molecules that participate in *Drosophila* Toll pathways, such as Dorsal, Cactus, or Pelle, had been previously identified by the isolation of the corresponding insect mutants in the 1980s [7]. In 1991, the amino acid sequence of the cytoplasmic portion of interleukin-1 (IL-1) was reported to resemble that of *Drosophila* Toll [8]. Since activation of IL-1R signaling pathways activated a transcription factor, nuclear factor kappa B (NF-*κ*B), a mammalian homolog of *Drosophila* Dorsal, the counterpart of *Drosophila* Toll had been first considered to be IL-1R at that time [8]. After identification of mammalian TLRs, TLRs and the IL-1R were demonstrated to possess a highly conserved intracellular domain, now known as the Toll-IL-1R (TIR) domain. Ligand recognition of TLRs causes dimerization of the cytoplasmic TIR domains, culminating in activation of downstream intracellular signaling. Similar to *Drosophila* Toll signaling and mammalian IL-1R signaling, TLR signaling also activates NF-*κ*B as well as mitogen-activated protein kinases (MAPKs) to stimulate gene expression, including proinflammatory cytokines and costimulatory molecules [9]. In addition, the mammalian TLR system establishes antiviral immune responses predominantly through the production of type I interferon (IFN) [10]. 

In this paper, we will discuss the current view of mammalian TLR pathways, focusing on the molecular basis of extracellular and intracellular signaling events. 

## 2. PAMPs and TLRs

So far, there are 10 members of the human and 13 members of the mouse TLR family that have been identified [1–3]. TLR1-TLR10 are conserved between humans and mice, although TLR10 is not functional in mice because of a retroviral insertion. In addition, TLR11-13 are not present in humans. Thus, despite some species-specific receptors, most members are conserved in mammals. 

Among the TLRs, the ligand of TLR4 was first identified by genetic studies. The C3H/HeJ mutant mouse strain is hyporesponsiveness to lipopolysaccharide (LPS), a cell wall component mainly found in Gram-negative bacteria, and possesses a recessive autosomal mutation in the Lps locus [11, 12]. Positional cloning of the Lps locus revealed a point mutation in the TLR4 gene. TLR4-deficient mice also showed a similar hyporesponsiveness to LPS [13]. C3H/HeJ-type TLR4 fails to activate NF-*κ*B in response to LPS, indicating that TLR4 is essential for the recognition of LPS *in vivo*. 

In addition to LPS, bacterial lipoprotein moieties are recognized by TLR1, TLR2, and TLR6. TLR1 plays an important role in the recognition of a synthetic lipoprotein, *N*-palmitoyl-*S*-dipalmitoylglyceryl (Pam_3_) Cys-Ser-(Lys)_4_ (CSK_4_) (Pam3CSK4), and the outer-surface lipoprotein of the pathogen *Borrelia burgdorferi*, outer surface protein A (OspA) [14, 15]. TLR6 participates in the recognition of macrophage-activating lipoprotein 2 kD (MALP-2) derived from mycoplasma [16]. Both TLR1 and TLR6 require dimerization with TLR2 to be functional [16]. TLR2 is also essential for the recognition of peptidoglycan, lipoarabinomannan, porins, *Trypanosoma cruzi* Glycosylphosphatidylinositol-anchored mucin-like glycoproteins (tGPI-mucin), or Hemagglutinin (HA) proteins from not only bacteria but also viruses or parasites [1]. 

Genomic nucleic acids from bacteria and viruses, or their analogs, stimulate the production of proinflammatory cytokines and type I IFN. Among them, immunostimulatory bacterial DNA was first identified in Calmette-Guerin bacilli, which are capable of promoting antitumor activity and inducing type I IFN (IFN-*α*/*β*) and type II IFN (IFN-*γ*) in human peripheral blood leukocytes [17, 18]. Among the TLR family members, TLR9 is responsible for the recognition of unmethylated CpG DNA. TLR9 also recognizes genomic DNA from DNA viruses such as HSV-1, HSV-2, or MCMV [1]. As well as nucleic acids, hemozoin, a malaria-derived insoluble crystal, is a ligand for TLR9 [19]. 

RNA is also a TLR ligand. TLR3 recognizes a synthetic double-stranded RNA (dsRNA) analog, polyinosinic-polycytidylic acid (poly I:C), and dsRNA derived from Reovirus, EMCV, RSV, or West Nile virus (WNV) [1]. In contrast to dsRNA recognition by TLR3, guanosine-rich and uridine-rich single-stranded RNAs (ssRNAs) derived from HIV or influenza virus are ligands for TLR7 [20, 21]. In addition, low molecular weight compounds of the nucleoside analog imidazoquinoline, known as Imiquimod (Aldara, R-837, S-26308), and R-848 (resiquimod, S-28463) are synthetic TLR7 ligands [22, 23]. 

**Figure 1:**
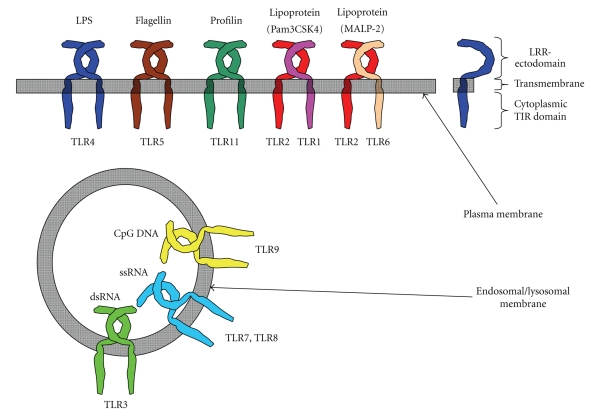
Extracellular and intracellular TLRs. TLRs can be divided into extracellular and intracellular TLRs. TLR1, TLR2, TLR4, TLR5, TLR6, and TLR11 recognize their ligands on the cell surface. On the other hand, TLR3, TLR7, TLR8, and TLR9 are intracellularly localized.

TLR5 and TLR11 recognize protein moieties from bacteria or parasites. TLR5 is essential for the recognition of a component of bacterial flagella, flagellin. The highly conserved central portion of flagellin that is pivotal for bacterial motility is bound by TLR5 [24]. TLR11 recognizes a parasite-derived profilin-like molecule that is a potent inducer of IL-12 and known as soluble Toxoplasma antigen. It plays an important role in parasite motility and invasion into host cells [25, 26]. TLR11 might also recognize PAMPs from uropathogenic bacteria, since TLR11-deficient mice are highly susceptible to the pathogen. However, the natural ligand for TLR11 from uropathogenic bacteria has not yet been identified [27]. Thus, TLRs recognize a number of PAMPs from various microbes. (See Figure 1).

## 3. Molecular Basis of TLRs Structure and Ligand Recognition

TLRs are type I transmembrane proteins that consist of three major domains: (1) a leucine rich extracellular domain; (2) a transmembrane domain; (3) a cytoplasmic TIR domain. Ligand recognition by TLRs is mediated by the extracellular domain that harbors a leucine rich repeat (LRR) composed of 19–25 tandem copies of the “xLxxLxLxx” motif [28]. So far, the crystal structure of TLR1, TLR2, TLR3, TLR4, and TLR6 with or without their ligands has been resolved, and these studies predict that the extracellular domain of the TLRs forms a horseshoe-like structure [28]. Notably, structural analysis and biochemical studies indicate that all TLRs form hetero- or homodimers (e.g., TLR1/TLR2, TLR2/TLR6, TLR3/TLR3, and TLR4/TLR4), which probably facilitates dimerization of the cytoplasmic TIR domain to activate intracellular signaling [28]. In the case of the homodimers of TLR3 or TLR4, direct or indirect interactions by ionic and hydrogen bonds with their ligands are essential for the recognition [29, 30]. On the other hand, TLR2 forms a heterodimer together with either TLR1 or TLR6 to recognize triacyl or diacyl peptides in internal protein pockets through hydrophilic interactions [31, 32].

## 4. Localization of TLRs: Extracellular and Intracellular TLRs

The TLR family can be largely divided into extracellular and intracellular members. TLR1, TLR2, TLR4, TLR5, TLR6, and TLR11 are largely localized on the cell surface to recognize PAMPs. On the other hand, TLR3, TLR4, TLR7, TLR8, and TLR9 are intracellularly expressed in endosomal- or lysosomal-compartments and the endoplasmic reticulum (ER). 

TLR4 is tightly bound to MD-2 on the cell surface [33]. In addition, CD14 and LPS-binding protein (LBP) also participate in the recognition of LPS by TLR4/MD-2. CD14 efficiently transfers an LBP-LPS complex to TLR4/MD-2 for cellular activation. In addition, TLR4 is internalized into endosomal compartments in response to LPS stimulation [33]. For TLR2 ligand recognition, TLR1, TLR6, and a host on non-TLR receptors, such as CD36 or Dectin-1, form heterodimers with TLR2 and are involved in the recognition of most TLR2 ligands or *β*-glucan, respectively [34, 35]. The intracellular TLRs such as TLR3, TLR7, TLR8, and TLR9 are localized on the ER membrane in resting cells. However, upon stimulation, they are trafficked to the endosomal compartment [36, 37]. The intracellular localization is regulated by the ER membrane protein UNC93B, which directly interacts with the intracellular TLRs [38]. Mice bearing a point missense mutation of this gene are defective in the trafficking of TLR3, TLR7, and TLR9 and fail to activate cellular signaling [38]. Moreover, processing of the ectodomain of TLR9 by cathepsins in the endolysosomal compartments is required for compartment-specific activation [39, 40]. Thus, the localization of TLRs responsible for detecting foreign (nonself) nucleic acids is tightly regulated to avoid a response to self-DNA on the cell surface [41]. (See Figure 1).

## 5. NF-*κ*B and MAPK Activation in TLR-Mediated Intracellular Signaling

As discussed above, the cytoplasmic portion of TLRs shows high similarity to that of IL-1R family members and is called the TIR domain. Activation of TLR signaling culminates in NF-*κ*B and MAPKs that regulate gene expression of various immune and inflammatory mediators [9]. In TLR signaling, NF-*κ*Bs are sequestered by cytoplasmic I*κ*B proteins including nuclear inhibitor of NF-*κ*B (I*κ*B)*α*, and I*κ*B*β* in unstimulated cells. Stimulation by TLR ligands mediates degradation of these I*κ*Bs by the proteasome, a process dependent on their phosphorylation by the I*κ*B kinase (IKK) complex composed of IKK*α*, IKK*β*, and NEMO (also known as IKK*γ*), allowing the nuclear translocation of NF-*κ*B [42]. Transcriptional activity is controlled by a variety of nuclear proteins such as Akirin2 or the family of nuclear I*κ*Bs, I*κ*B*ζ*, I*κ*BNS, and Bcl-3 [43–45].

The activation of the IKK complex is regulated by several MAPK kinase kinases (MAP3K) including TAK1, MEKK3, Tpl2, and ASK1 [1]. Among them, TAK1 has been most studied in view of its molecular role in TLR/IL-1R-mediated IKK activation [46]. TAK1 participates in IKK activation in a complex with TAK1-binding protein 1 (TAB1), TAB2, and TAB3, at least *in vitro* [46]. However, the roles of TAB protein family members in TLR/IL-1R-mediated signaling remain controversial, since neither TAB1 nor TAB2-deficient mice showed any abnormalities of TLR/IL-1R signaling pathways [47–49]. The activation of the TAK1-TABs complex is regulated not by protein phosphorylation, but by lysine 63 (K63)-linked ubiquitination [50]. In contrast to K48-linked ubiquitination that mediates proteasome-dependent protein destruction, K63-linked ubiquitination is involved in cellular processes such as DNA repair, in addition to activation of the TAK1-TABs complex [51]. The formation of K63-linked polyubiquitin chains is catalyzed by the E2 ubiquitin conjugating enzyme complex Ubc13 and Uev1A [52]. The role of Ubc13 in TLR-mediated NF-*κ*B activation remains controversial, since mice lacking Ubc13 exhibit almost normal NF-*κ*B activation and I*κ*B degradation in response to TLR ligands or IL-1 [53]. The TAK1 complex also regulates activation of MAPKs, such as ERK1/2, p38, and JNK, to control mRNA expression or the stability of mRNA for inflammatory genes by mediating phosphorylation of AP-1 transcription family proteins [54, 55].

**Figure 2:**
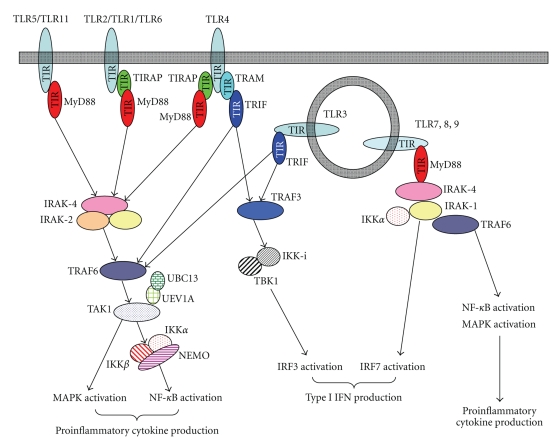
TLR-mediated MyD88-dependent or TRIF-dependent pathways. TIR domain-containing adaptors MyD88, TIRAP, TRIF, and TRAM define TLR-mediated signaling. MyD88 and TIRAP are adaptors for the MyD88-dependent pathways, which mainly activate proinflammatory cytokine production. On the other hand, TRIF and TRAM are adaptors for IRF3 activation, resulting in production of type I IFN by TLR3- or TLR4-mediated TRIF-dependent pathways. In pDCs, TLR7/TLR9-mediated MyD88-dependent pathways induce IRF7 activation, leading to IFN*α* production.

Upstream of the TAK1 complex, a RING finger-containing E3 ligase, TRAF6, is involved in K63-linked ubiquitination-mediated signaling [1]. The activity of TRAF6 is regulated by the family of death domain containing-IL-1R-associated kinases (IRAKs), IRAK1, IRAK2, IRAK-M, and IRAK-4 [56, 57]. Among them, IRAK-1, IRAK-2, and IRAK-4 positively regulate TRAF6 activity, while IRAK-M limits TLR/IL-1R-medaited immune responses. Genetic studies using mice lacking IRAK-1, IRAK-2 or IRAK-4 have demonstrated that deficiency of both IRAK-1 and IRAK-2 is comparable to the lack of IRAK-4 alone, suggesting a central role of IRAK-4 in TLR/IL-1R-mediated signaling. Moreover, IRAK-4 interacts with an upstream adaptor molecule, MyD88, through a homophilic interaction of the death domain [58]. MyD88 plays a critical role in both TLR- and IL-1R-mediated signaling pathways [59, 60]. (See Figure 2).

## 6. Specific Participation of the TIR Domain-Containing Adaptor Molecules in TLR Signaling

MyD88 is a member of the family of cytosolic TIR domain-containing adaptor molecules. In addition to MyD88, the family includes TIRAP (also known as Mal), TRIF (also known as TICAM-1), TRAM (also known as TICAM-2), and SARM. As discussed above, the cytoplasmic portion of TLRs harbors a TIR domain, to which individual TIR domain-containing adaptors are selectively recruited to specific TLRs, generating signaling specificity for each TLR [9]. MyD88 is a master adaptor molecule that is utilized by not only all IL-1R family members, but also by almost all TLRs, with the exception of TLR3 [1]. TIRAP interacts with MyD88 through the TIR domain and selectively participates in TLR2- and TLR4-mediated MyD88-dependent signaling pathways [61, 62]. 

Whereas LPS, the TLR4 ligand, fails to stimulate the production of proinflammatory cytokines in MyD88-deficient cells, it still activates NF-*κ*B and MAPK and induces gene expression of type I IFN, indicating the presence of MyD88-independent pathways in TLR4 signaling [63, 64]. Moreover, TLR3 activates MyD88-independent signaling, suggesting the existence of other TIR domain-containing adaptor molecules that function in the TLR3- and TLR4-mediated pathways [65]. TRIF plays a critical role in the TLR3- and TLR4-mediated MyD88-independent pathways [63, 64]. Although TRIF is bound to TLR3 through the TIR domain, TLR4 utilizes TRAM to activate TRIF-dependent signaling [66–68]. Thus, TLR4 utilizes MyD88 and TIRAP for the MyD88-dependent pathway to induce mainly proinflammatory cytokines, or TRIF and TRAM for the MyD88-independent pathway to induce type I IFN and IFN inducible genes (IRGs) [9]. Moreover, internalization of TLR4 is shown to be required for proper activation of the TRIF-dependent pathway [69]. In addition, a very recent study demonstrates a two-stage activation mechanism for TLR4-mediated signaling pathways, in which assembly of a multiprotein complex including MyD88, TRAF6, Ubc13, IKK*γ*, cIAP1/2, TAK1, and TRAF3 induces K63-linked ubiquitination of cIAP1/2 that leads to degradation of TRAF3, subsequently resulting in MyD88-signaling complex inducing its translocation from membrane to the cytosol and TAK1 activation [70].

Regarding SARM, a previous biochemical report has suggested that human SARM is required for negative regulation of the TLR3-mediated MyD88-independent pathway by inhibiting the interaction of TRIF with TLR3 [71]. However, mice lacking SARM do not show any abnormalities in TLR3-mediated, or other TLR-mediated, signaling, indicating the minor role of SARM in TLR signaling pathways [72].

## 7. Signaling Pathways for TLR-Mediated Type I IFN Production

TLR3/TLR4-mediated MyD88-independent (TRIF-dependent) signaling induces the expression of type I IFN and IRGs [9]. In addition, TLR7- and TLR9-mediated MyD88-dependent pathways also lead to type I IFN production, especially in a subtype of dendritic cells called plasmacytoid dendritic cells (pDCs) [73]. Thus, TLR-mediated type I IFN production is mediated by the TLR3/TLR4-mediated TRIF-dependent or the TLR7/TLR9-mediated MyD88-dependent pathways [1]. Expression of type I IFN is largely controlled by IRF transcription factors, which consist of 9 members. Among them, IRF1, IRF3, IRF7, and IRF8 are involved in TLR-mediated type I IFN production [74].

Downstream of TRIF, two TRAF proteins, TRAF3 and TRAF6, participate in type I IFN production [75, 76]. Although deficiency in TRAF3 has resulted in severe abnormalities in TRIF-mediated type I IFN production as well as IL-10 production, it is largely normal in TRAF6-deficient cells, suggesting that TRAF3 rather than TRAF6 plays the critical role in the TLR3/TLR4-mediated TRIF-dependent pathway [75, 76]. Signaling from TRAF3 activates IRF3, predominantly leading to IFN-*β* expression [75, 76]. Phosphorylation of the C-terminal serine/threonine rich portions of IRF3 by IKK-related kinases, TBK1 (also known as T2K or NAK) and IKK-*i* (also known as IKK*ε*), is required for this activation [77, 78]. Phosphorylated IRF3 forms a homodimer and translocates into the nucleus where it binds to the DNA and interacts with the nuclear coactivator proteins p300 and CBP to positively regulate the transcription of the IFN*β* gene [79]. IKK-*i* and TBK1 are bound to TANK (also known as I-TRAF), NAP1 (also known as AZ2) and TBKBP1 (also known as SINTBAD) [80–82]. Although the molecules regulate the kinase activities of TBK1 and IKK-*i*, much like NEMO, which critically controls IKK*α* and IKK*β*, their physiological roles in TLR3/TLR4-mediated type I IFN production remain to be elucidated [1]. 

*In vivo* stimulation by TLR7 or TLR9 ligands in mice leads to the production of high levels of IFN*α*, mainly from pDCs [1]. TLR7- and TLR9-dependent IFN*α* production requires MyD88, but not TRIF [83, 84]. MyD88-dependent expression of IFN*α* is mediated by IRF7 [83, 84], a protein structurally related to IRF3 that plays a master role in TLR7- and TLR9-mediated IFN*α* production [74]. Like IRF3, which is phosphorylated by IKK-*i* and TBK1 [85, 86], IRF7 is also phosphorylated in response to TLR7 and TLR9 ligands in pDCs, although by IRAK-1 and IKK*α* [83]. Moreover, PI3K, osteopontin, TRAF3, and TRAF6 regulate IRF7 activation [87]. In addition to IRF7, IRF1 participates in IFN*β* gene expression in conventional DCs (cDCs) [88]. IRF1 as well as IRF7 is directly bound to MyD88 and activated in response to TLR9 ligands [89]. Moreover, IRF8 also plays an important role in IFN*α* and IFN*β*, as well as IL-12 p40, production by pDCs and other DCs [90]. Another IRF member, IRF5, is indispensable for proinflammatory cytokine production, rather than type I IFN, in the MyD88-dependent pathways mediated by almost all TLRs [91]. Thus, IRFs are divergent regulators of the production of not only type I IFN, but also inflammatory cytokines. (See Figure 2).

## 8. Conclusions

A variety of PAMPs derived from a wide range of pathogens, including viruses, bacteria, fungi, and parasites, are recognized by TLRs. After ligation of the PAMPs, TLRs initiate intracellular signaling pathways to activate immune responses via the TIR domain. The TIR domain of the receptor interacts with the intracellular TIR domain-containing adaptors MyD88, TIRAP, TRIF, TRAM, and SARM, which generate the specificity of the downstream signaling and unique outputs of each TLR. The MyD88-dependent pathways mainly regulate proinflammatory cytokine production and IRF1- or IRF7-mediated type I IFN production in DCs. On the other hand, TLR3- and TLR4-mediated TRIF-dependent pathways control IRF3-mediated IFN*β* production. Large bodies of the past, current, and future literature on TLR signaling pathways can be applied not only to liver immune cells, such as Kupffer cells, but also nonimmune cells including hepatocytes and other liver cells.

## References

[1] Akira S, Uematsu S, Takeuchi O (2006). Pathogen recognition and innate immunity. *Cell*.

[2] Beutler B, Eidenschenk C, Crozat K (2007). Genetic analysis of resistance to viral infection. *Nature Reviews Immunology*.

[3] Medzhitov R (2007). Recognition of microorganisms and activation of the immune response. *Nature*.

[4] Janeway CA (1989). Approaching the asymptote? Evolution and revolution in immunology. *Cold Spring Harbor Symposia on Quantitative Biology*.

[5] Lemaitre B, Nicolas E, Michaut L, Reichhart J-M, Hoffmann JA (1996). The dorsoventral regulatory gene cassette spatzle/Toll/cactus controls the potent antifungal response in *Drosophila* adults. *Cell*.

[6] Medzhitov R, Preston-Hurlburt P, Janeway CA (1997). A human homologue of the *Drosophila* toll protein signals activation of adaptive immunity. *Nature*.

[7] Hoffmann JA (2003). The immune response of *Drosophila*. *Nature*.

[8] Gay NJ, Keith FJ (1991). Drosophila Toll and IL-1 receptor. *Nature*.

[9] Yamamoto M, Takeda K, Akira S (2004). TIR domain-containing adaptors define the specificity of TLR signaling. *Molecular Immunology*.

[10] Kawai T, Akira S (2006). Innate immune recognition of viral infection. *Nature Immunology*.

[11] Poltorak A, He X, Smirnova I (1998). Defective LPS signaling in C3H/HeJ and C57BL/10ScCr mice: mutations in Tlr4 gene. *Science*.

[12] Qureshi ST, Larivière L, Leveque G (1999). Endotoxin-tolerant mice have mutations in toll-like receptor 4 (Tlr4). *Journal of Experimental Medicine*.

[13] Hoshino K, Takeuchi O, Kawai T (1999). Cutting edge: Toll-like receptor 4 (TLR4)-deficient mice are hyporesponsive to lipopolysaccharide evidence for TLR4 as the Lps gene product. *Journal of Immunology*.

[14] Takeuchi O, Sato S, Horiuchi T (2002). Cutting edge: role of Toll-like receptor 1 in mediating immune response to microbial lipoproteins. *Journal of Immunology*.

[15] Alexopoulou L, Thomas V, Schnare M (2002). Hyporesponsiveness to vaccination with Borrelia burgdorferi OspA in humans and in TLR1- and TLR2-deficient mice. *Nature Medicine*.

[16] Takeuchi O, Kawai T, Mühlradt PF (2001). Discrimination of bacterial lipoproteins by Toll-like recepttor 6. *International Immunology*.

[17] Wagner H (2002). Interactions between bacterial CpG-DNA and TLR9 bridge innate and adaptive immunity. *Current Opinion in Microbiology*.

[18] Krieg AM (2002). CpG motifs in bacterial DNA and their immune effects. *Annual Review of Immunology*.

[19] Coban C, Ishii KJ, Kawai T (2005). Toll-like receptor 9 mediates innate immune activation by the malaria pigment hemozoin. *Journal of Experimental Medicine*.

[20] Diebold SS, Kaisho T, Hemmi H, Akira S, Reis E Sousa C (2004). Innate antiviral responses by means of TLR7-mediated recognition of single-stranded RNA. *Science*.

[21] Heil F, Hemmi H, Hochrein H (2004). Species-specific recognition of single-stranded RNA via toll-like receptor 7 and 8. *Science*.

[22] Miller RL, Gerster JF, Owens ML, Slade HB, Tomai MA (1999). Review article Imiquimod applied topically: a novel immune response modifier and new class of drug. *International Journal of Immunopharmacology*.

[23] Tyring S (1998). Immune response modification: imiquimod. *Australasian Journal of Dermatology*.

[24] Hayashi F, Smith KD, Ozinsky A (2001). The innate immune response to bacterial flagellin is mediated by Toll-like receptor 5. *Nature*.

[25] Plattner F, Yarovinsky F, Romero S (2008). Toxoplasma profilin is essential for host cell invasion and TLR11-dependent induction of an interleukin-12 response. *Cell Host and Microbe*.

[26] Yarovinsky F, Zhang D, Andersen JF (2005). Immunology: TLR11 activation of dendritic cells by a protozoan profilin-like protein. *Science*.

[27] Zhang D, Zhang G, Hayden MS (2004). A Toll-like receptor that prevents infection by uropathogenic bacteria. *Science*.

[28] Jin MS, Lee J-O (2008). Structures of the Toll-like receptor family and its ligand complexes. *Immunity*.

[29] Choe J, Kelker MS, Wilson IA (2005). Structural biology: crystal structure of human toll-like receptor 3 (TLR3) ectodomain. *Science*.

[30] Kim HM, Park BS, Kim J-I (2007). Crystal structure of the TLR4-MD-2 complex with bound endotoxin antagonist Eritoran. *Cell*.

[31] Jin MS, Kim SE, Heo JY (2007). Crystal structure of the TLR1-TLR2 heterodimer induced by binding of a tri-acylated lipopeptide. *Cell*.

[32] Kang JY, Nan X, Jin MS (2009). Recognition of lipopeptide patterns by Toll-like receptor 2-Toll-like receptor 6 heterodimer. *Immunity*.

[33] Miyake K (2007). Innate immune sensing of pathogens and danger signals by cell surface Toll-like receptors. *Seminars in Immunology*.

[34] Hoebe K, Georgel P, Rutschmann S (2005). CD36 is a sensor of diacylglycerides. *Nature*.

[35] Gantner BN, Simmons RM, Canavera SJ, Akira S, Underhill DM (2003). Collaborative induction of inflammatory responses by dectin-1 and toll-like receptor 2. *Journal of Experimental Medicine*.

[36] Latz E, Schoenemeyer A, Visintin A (2004). TLR9 signals after translocating from the ER to CpG DNA in the lysosome. *Nature Immunology*.

[37] Nishiya T, Kajita E, Miwa S, DeFranco AL (2005). TLR3 and TLR7 are targeted to the same intracellular compartments by distinct regulatory elements. *Journal of Biological Chemistry*.

[38] Tabeta K, Hoebe K, Janssen EM (2006). The Unc93b1 mutation 3d disrupts exogenous antigen presentation and signaling via Toll-like receptors 3, 7 and 9. *Nature Immunology*.

[39] Ewald SE, Lee BL, Lau L (2008). The ectodomain of Toll-like receptor 9 is cleaved to generate a functional receptor. *Nature*.

[40] Park B, Brinkmann MM, Spooner E, Lee CC, Kim Y-M, Ploegh HL (2008). Proteolytic cleavage in an endolysosomal compartment is required for activation of Toll-like receptor 9. *Nature Immunology*.

[41] Barton GM, Kagan JC, Medzhitov R (2006). Intracellular localization of Toll-like receptor 9 prevents recognition of self DNA but facilitates access to viral DNA. *Nature Immunology*.

[42] Karin M, Yamamoto Y, Wang QM (2004). The IKK NF-*κ*B system: a treasure trove for drug development. *Nature Reviews Drug Discovery*.

[43] Yamamoto M, Yamazaki S, Uematsu S (2004). Regulation of Toll/IL-1-receptor-mediated gene expression by the inducible nuclear protein I*κ*B*ζ*. *Nature*.

[44] Goto A, Matsushita K, Gesellchen V (2008). Akirins are highly conserved nuclear proteins required for NF-*κ*B-dependent gene expression in drosophila and mice. *Nature Immunology*.

[45] Yamamoto M, Takeda K (2008). Role of nuclear I*κ*B proteins in the regulation of host immune responses. *Journal of Infection and Chemotherapy*.

[46] Delaney JR, Mlodzik M (2006). TGF-*β* activated kinase-1: new insights into the diverse roles of TAK1 in development and immunity. *Cell Cycle*.

[47] Komatsu Y, Shibuya H, Takeda N (2002). Targeted disruption of the Tab1 gene causes embryonic lethality and defects in cardiovascular and lung morphogenesis. *Mechanisms of Development*.

[48] Sanjo H, Takeda K, Tsujimura T, Ninomiya-Tsuji J, Matsumoto K, Akira S (2003). TAB2 is essential for prevention of apoptosis in fetal liver but not for interleukin-1 signaling. *Molecular and Cellular Biology*.

[49] Inagaki M, Komatsu Y, Scott G (2008). Generation of a conditional mutant allele for Tab1 in mouse. *Genesis*.

[50] Adhikari A, Xu M, Chen ZJ (2007). Ubiquitin-mediated activation of TAK1 and IKK. *Oncogene*.

[51] Hofmann RM, Pickart CM (1999). Noncanonical MMS2-encoded ubiquitin-conjugating enzyme functions in assembly of novel polyubiquitin chains for DNA repair. *Cell*.

[52] Kawai T, Akira S (2007). TLR signaling. *Seminars in Immunology*.

[53] Yamamoto M, Okamoto T, Takeda K (2006). Key function for the Ubc13 E2 ubiquitin-conjugating enzyme in immune receptor signaling. *Nature Immunology*.

[54] Sato S, Sanjo H, Takeda K (2005). Essential function for the kinase TAK1 in innate and adaptive immune responses. *Nature Immunology*.

[55] Shim J-H, Xiao C, Paschal AE (2005). TAK1, but not TAB1 or TAB2, plays an essential role in multiple signaling pathways in vivo. *Genes and Development*.

[56] Janssens S, Beyaert R (2003). Functional diversity and regulation of different interleukin-1 receptor-associated kinase (IRAK) family members. *Molecular Cell*.

[57] Suzuki N, Suzuki S, Yeh W-C (2002). IRAK-4 as the central TIR signaling mediator in innate immunity. *Trends in Immunology*.

[58] Li S, Strelow A, Fontana EJ, Wesche H (2002). IRAK-4: a novel member of the IRAK family with the properties of an IRAK-kinase. *Proceedings of the National Academy of Sciences of the United States of America*.

[59] Adachi O, Kawai T, Takeda K (1998). Targeted disruption of the MyD88 gene results in loss of IL-1- and IL- 18-mediated function. *Immunity*.

[60] Takeuchi O, Kaufmann A, Grote K (2000). Cutting edge: preferentially the R-stereoisomer of the mycoplasmal lipopeptide macrophage-activating lipopeptide-2 activates immune cells through a toll-like receptor 2- and MyD88-dependent signaling pathway. *Journal of Immunology*.

[61] Yamamoto M, Sato S, Hemmi H (2002). Essential role for TIRAP in activation of the signalling cascade shared by TLR2 and TLR4. *Nature*.

[62] Horng T, Barton GM, Flavell RA, Medzhitov R (2002). The adaptor molecule TIRAP provides signalling specificity for Toll-like receptors. *Nature*.

[63] Yamamoto M, Sato S, Hemmi H (2003). Role of adaptor TRIF in the MyD88-independent toll-like receptor signaling pathway. *Science*.

[64] Hoebe K, Du X, Georgel P (2003). Identification of Lps2 as a key transducer of MyD88-independent TIR signalling. *Nature*.

[65] Alexopoulou L, Holt AC, Medzhitov R, Flavell RA (2001). Recognition of double-stranded RNA and activation of NF-*κ*B by Toll-like receptor 3. *Nature*.

[66] Yamamoto M, Sato S, Hemmi H (2003). TRAM is specifically involved in the Toll-like receptor 4-mediated MyD88-independent signaling pathway. *Nature Immunology*.

[67] Fitzgerald KA, Rowe DC, Barnes BJ (2003). LPS-TLR4 signaling to IRF-3/7 and NF-*κ*B involves the toll adapters TRAM and TRIF. *Journal of Experimental Medicine*.

[68] Oshiumi H, Sasai M, Shida K, Fujita T, Matsumoto M, Seya T (2003). TIR-containing adapter molecule (TICAM)-2, a bridging adapter recruiting to toll-like receptor 4 TICAM-1 that induces interferon-*β*. *Journal of Biological Chemistry*.

[69] Kagan JC, Su T, Horng T, Chow A, Akira S, Medzhitov R (2008). TRAM couples endocytosis of Toll-like receptor 4 to the induction of interferon-*β*. *Nature Immunology*.

[70] Tseng P-H, Matsuzawa A, Zhang W, Mino T, Vignali DAA, Karin M (2010). Different modes of ubiquitination of the adaptor TRAF3 selectively activate the expression of type I interferons and proinflammatory cytokines. *Nature Immunology*.

[71] Carty M, Goodbody R, Schröder M, Stack J, Moynagh PN, Bowie AG (2006). The human adaptor SARM negatively regulates adaptor protein TRIF-dependent Toll-like receptor signaling. *Nature Immunology*.

[72] Kim Y, Zhou P, Qian L (2007). MyD88-5 links mitochondria, microtubules, and JNK3 in neurons and regulates neuronal survival. *Journal of Experimental Medicine*.

[73] Gilliet M, Cao W, Liu Y-J (2008). Plasmacytoid dendritic cells: sensing nucleic acids in viral infection and autoimmune diseases. *Nature Reviews Immunology*.

[74] Honda K, Taniguchi T (2006). IRFs: master regulators of signalling by Toll-like receptors and cytosolic pattern-recognition receptors. *Nature Reviews Immunology*.

[75] Häcker H, Redecke V, Blagoev B (2006). Specificity in Toll-like receptor signalling through distinct effector functions of TRAF3 and TRAF6. *Nature*.

[76] Oganesyan G, Saha SK, Guo B (2006). Critical role of TRAF3 in the Toll-like receptor-dependent and -independent antiviral response. *Nature*.

[77] Fitzgerald KA, McWhirter SM, Faia KL (2003). IKKE and TBKI are essential components of the IRF3 signalling pathway. *Nature Immunology*.

[78] Sharma S, Ten Oever BR, Grandvaux N, Zhou G-P, Lin R, Hiscott J (2003). Triggering the interferon antiviral response through an IKK-related pathway. *Science*.

[79] Yoneyama M, Suhara W, Fukuhara Y, Fukuda M, Nishida E, Fujita T (1998). Direct triggering of the type I interferon system by virus infection: activation of a transcription factor complex containing IRF-3 and CBP/p300. *EMBO Journal*.

[80] Fujita F, Taniguchi Y, Kato T (2003). Identification of NAP1, a Regulatory Subunit of I*κ*B Kinase-Related Kinases That Potentiates NF-*κ*B Signaling. *Molecular and Cellular Biology*.

[81] Ryzhakov G, Randow F (2007). SINTBAD, a novel component of innate antiviral immunity, shares a TBK1-binding domain with NAP1 and TANK. *EMBO Journal*.

[82] Sasai M, Shingai M, Funami K (2006). NAK-associated protein 1 participates in both the TLR3 and the cytoplasmic pathways in type I IFN induction. *Journal of Immunology*.

[83] Kawai T, Sato S, Ishii KJ (2004). Interferon-*α* induction through Toll-like receptors involves a direct interaction of IRF7 with MyD88 and TRAF6. *Nature Immunology*.

[84] Honda K, Yanai H, Negishi H (2005). IRF-7 is the master regulator of type-I interferon-dependent immune responses. *Nature*.

[85] Uematsu S, Sato S, Yamamoto M (2005). Interleukin-1 receptor-associated kinase-1 plays an essential role for Toll-like receptor (TLR)7- and TLR9-mediated interferon-*α* induction. *Journal of Experimental Medicine*.

[86] Hoshino K, Sugiyama T, Matsumoto M (2006). I*κ*B kinase-*α* is critical for interferon-*α* production induced by Toll-like receptors 7 and 9. *Nature*.

[87] Kawai T, Akira S (2009). The roles of TLRs, RLRs and NLRs in pathogen recognition. *International Immunology*.

[88] Negishi H, Fujita Y, Yanai H (2006). Evidence for licensing of IFN-*γ*-induced IFN regulatory factor 1 transcription factor by MyD88 in Toll-like receptor-dependent gene induction program. *Proceedings of the National Academy of Sciences of the United States of America*.

[89] Schmitz F, Heit A, Guggemoos S (2007). Interferon-regulatory-factor 1 controls Toll-like receptor 9-mediated IFN-*β* production in myeloid dendritic cells. *European Journal of Immunology*.

[90] Tailor P, Tamura T, Kong HJ (2007). The feedback phase of type I interferon induction in dendritic cells requires interferon regulatory factor 8. *Immunity*.

[91] Takaoka A, Yanai H, Kondo S (2005). Integral role of IRF-5 in the gene induction programme activated by Toll-like receptors. *Nature*.

